# Interactions between Urinary 4-tert-Octylphenol Levels and Metabolism Enzyme Gene Variants on Idiopathic Male Infertility

**DOI:** 10.1371/journal.pone.0059398

**Published:** 2013-03-15

**Authors:** Yufeng Qin, Minjian Chen, Wei Wu, Bin Xu, Rong Tang, Xiaojiao Chen, Guizhen Du, Chuncheng Lu, John D. Meeker, Zuomin Zhou, Yankai Xia, Xinru Wang

**Affiliations:** 1 State Key Laboratory of Reproductive Medicine, Institute of Toxicology, Nanjing Medical University, Nanjing, China; 2 Key Laboratory of Modern Toxicology of Ministry of Education, School of Public Health, Nanjing Medical University, Nanjing, China; 3 Department of Environmental Health Sciences, University of Michigan School of Public Health, Ann Arbor, Michigan, United States of America; University of Hawaii at Manoa, John A. Burns School of Medicine, United States of America

## Abstract

Octylphenol (OP) and Trichlorophenol (TCP) act as endocrine disruptors and have effects on male reproductive function. We studied the interactions between 4-tert-Octylphenol (4-t-OP), 4-n- Octylphenol (4-n-OP), 2,3,4-Trichlorophenol (2,3,4-TCP), 2,4,5-Trichlorophenol (2,4,5-TCP) urinary exposure levels and polymorphisms in selected xenobiotic metabolism enzyme genes among 589 idiopathic male infertile patients and 396 controls in a Han-Chinese population. Ultra high performance liquid chromatography-tandem mass spectrometry (UPLC-MS/MS) was used to measure alkylphenols and chlorophenols in urine. Polymorphisms were genotyped using the SNPstream platform and the Taqman method. Among four phenols that were detected, we found that only exposure to 4-t-OP increased the risk of male infertility (*P*_trend_ = 1.70×10^−7^). The strongest interaction was between 4-t-OP and rs4918758 in *CYP2C9* (*P*_inter_ = 6.05×10^−7^). It presented a significant monotonic increase in risk estimates for male infertility with increasing 4-t-OP exposure levels among men with TC/CC genotype (low level compared with non-exposed, odds ratio (OR) = 2.26, 95% confidence intervals (CI) = 1.06, 4.83; high level compared with non-exposed, OR = 9.22, 95% CI = 2.78, 30.59), but no associations observed among men with TT genotype. We also found interactions between 4-t-OP and rs4986894 in CYP2C19, and between rs1048943 in CYP1A1, on male infertile risk (*P*_inter_ = 8.09×10^−7^, *P*_inter_ = 3.73×10^−4^, respectively).We observed notable interactions between 4-t-OP exposure and metabolism enzyme gene polymorphisms on idiopathic infertility in Han-Chinese men.

## Introduction

Octylphenol (OP) and Trichlorophenol (TCP) are the representative members of alkylphenols and chlorophenols, chemicals that are extensively used in a wide range of industrial, commercial and agricultural applications [1]–[3]. In recent years, health concerns related to their widespread use have surfaced based on evidence for bioaccumulation in organisms and reproductive toxicity [4], [5]. Two isoforms of OP, 4-tert-Octylphenol (4-t-OP) and 4-n-Octylphenol (4-n-OP) are still used in Asian countries and are commonly detected in environmental media, such as wastewaters, fish tissue and are ubiquitous in the food supply [2], [6]. Exposure to OP is a matter of concern because it has been shown to be estrogenic and has the potential to interfere with normal endocrine function through human estrogen receptors [7]. Toxicological studies have consistently reported associations between exposure to OPs and impaired health, including adverse effects on male reproduction [8]–[10]. 2,3,4-Trichlorophenol (2,3,4-TCP) and 2,4,5-Trichlorophenol (2,4,5-TCP) are important representatives of chlorinated organic compounds. Besides presenting human health risks through oral exposure, TCP tends to bioaccumulate following release into the environment during the manufacture of chlorinated compounds and during the chlorination of drinking water [11]–[13]. Therefore, exposure to TCPs can occur through ingestion of food and water. A recent study indicated that TCPs may reduce testosterone levels by disrupting cAMP signaling [14].

Human exposure to estrogenic chemicals is hypothesized to be associated with a range of reproductive disorders, such as testicular germ line cancer and prostate intraepithelial hyperplasias [15]–[17]. However, these xenobiotics can be detoxified and the enzymes involved in this process include phase I enzymes, e.g., cytochrome P450 (CYP) family, and phase II enzymes, e.g., N-acetyltransferases (NATs) and sulfotransferases (SULTs) [18], [19]. Therefore, the activity of these enzymes may modify the process of activation and/or detoxification of chemicals. In addition, it has been reported that exposure to phenols may inhibit metabolism enzyme activity [20]. Genetic variants can also modify the catalytic activity of enzymes [21], [22]. Thus, susceptibility to the effects of phenols exposure may vary between individuals, possibly due to polymorphisms in metabolizing enzymes. To our knowledge, no studies have investigated the potential interaction between OPs and TCPs exposure and metabolism enzyme genes variants in relation to male infertility in Han-Chinese population.

In this study, we assessed relationships between urinary concentrations of several OPs and TCPs and male infertility for the first time. We also studied the potential interaction of OPs, TCPs and variants in metabolism enzyme genes on male infertility.

## Materials and Methods

### Study Population and Sample Collection

The protocol and consent form were approved by the Institutional Review Board of Nanjing Medical University prior to the study. Fertile controls and male infertility patients were consecutively recruited from the Affiliated Hospitals of Nanjing Medical University from 2005 to 2008. The patients had been unable to conceive for at least 12 months (without diagnosed infertile wives) and had undergone a complete historical and physical examination. The control subjects were fertile men from the early pregnancy registry of the same hospitals who were in the third month following a successful pregnancy. They were healthy men with normal reproductive function and confirmed having healthy babies 6–8 months later [23]. All activities involving human subjects were done under full compliance with government policies and the Helsinki Declaration. Consecutive eligible men were recruited to participate, 1317 in total were asked. Of those approached, 89.2% consented (1175 participants; 707 cases and 468 controls). After the study procedures were explained and all questions were answered, subjects signed informed consent forms. A completed physical examination including height and weight was performed, and a questionnaire was used to collect information including personal background, lifestyle factors, occupational and environmental exposures, genetic risk factors, sexual and reproduction status, medical history and physical activity. Men with abnormal sexual and ejaculatory functions, immune infertility, semen non-liquefaction, medical history of risk factors for infertility (e.g., varicocele, postvasectomy or orchidopexy), and receiving treatment for infertility (e.g., hormonal treatments) were excluded from the study (54 of 707 subjects). Men with other known causes related to male infertility, such as genetic disease, infection, or occupational exposure to agents suspected to be associated with male reproduction, were also excluded (34 of 653 subjects). Furthermore, to avoid azoospermia or severe oligozoospermia caused by Y chromosome microdeletions, we excluded subjects with Y chromosome microdeletions of Azoospermia Factor (AZF) region (11 of 619 subjects, microdeletion rate was 1.78%). Those subjects who declined to leave both blood samples and urine samples were excluded in our study. We also excluded the samples that did not pass quality control checks. In total 396 fertile controls and 589 infertile patients were included in this study and claimed that their life styles and environments had not changed for several months leading up to sample collection. After completing a questionnaire including detailed information, such as age, cigarette smoking, and alcohol consumption, each subject donated 5 ml of blood which was used for genomic DNA extraction and a urine sample for measuring the concentrations of 4-t-OP, 4-n-OP, 2,3,4-TCP and 2,4,5-TCP.

### Exposure Assessment

We measured total urinary concentrations of 4-t-OP, 4-n-OP, 2,3,4-TCP and 2,4,5-TCP using ultra high performance liquid chromatography-tandem mass spectrometry (UPLC-MS/MS). Briefly, urine samples were incubated in 1 M ammonium acetate buffer solution (pH = 5.0) for hydrolyzation with β-glucuronidase/sulfatase (20000 units/mL) overnight. After hydrolysis, the phenols were extracted and preconcentrated with solid phase extraction (500 mg/3 mL, Supelclean, USA), and determined with UPLC (Acquity UPLCTM 6 BEH C18 column, 1.7 µm, 2.1×100 mm) electrospray ionization (negative ion mode)-MS/MS. The limits of detection (LOD) were 0.28 µg/L (2,3,4-TCP), 0.15 µg/L (2,4,5-TCP), 0.34 µg/L (4-t-OP) and 0.02 µg/L (4-n-OP). Creatinine concentrations were analyzed using an automated chemistry analyzer (7020 Hitachi, Tokyo, Japan).

### SNP Selection and Genotype Analyses

Through information gained from PubMed and Hapmap, we identified twenty potential functional polymorphisms in metabolism enzymes: *CYP1A1* rs1048943, *CYP2B6* rs3760657, rs2054675, rs707265 and rs1042389, *CYP2C8* rs1058932, *CYP2C9* rs4918758, *CYP2C19* rs3814637, rs4986894 and rs11568732, *CYP2S1* rs3810171 and rs338583, *NAT1* rs7845127and rs10888150, *NAT2* rs1799930, rs1799931, rs4646246 and rs4646243, *SULT1E1* rs4149525 and rs3736599 (Table 1). All selected single nucleotide polymorphism (SNPs) have reported minor allele frequencies (MAF) of >0.05 in Han-Chinese population and are located in potential functional areas. In the case of multiple SNPs in the same haplotype block (linkage coefficient r^2^>0.8), only one was selected. Eight SNPs (rs2054675, rs707265, rs1042389, rs4986894, rs11568732, rs338583, rs10888150, rs1799931) were genotyped by using TaqMan SNP Genotyping Assays (Biosteed, Nanjing, China) and the other twelve SNPs (rs1048943, rs3760657, rs1058932, rs4918758, rs3814637, rs3810171, rs7845127, rs1799930, rs4646246, rs4646243, rs4149525 and rs3736599) were genotyped by GenomeLab SNPstream high throughput 12-plex genotyping Platform (Beckman Coulter, Fullerton, CA) following the manufacturer’s instructions [24]. For quality control, 10% of the samples were randomly genotyped again, and the repeatability was 100%.

**Table 1:** Characteristics of the cases and controls.

Variable	Controls (n = 396)	Cases (n = 589)
Age(years, mean ± SD)	29.75±3.43	28.41±4.61^a^
Smoking status		
Ever	189 (52.1)	306 (52.4)
Never	174 (47.9)	278 (47.6)
Drinking		
Yes	184 (51.0)	299 (51.1)
No	177 (49.0)	286 (48.9)
BMI	23.73±3.33	23.26±3.19^a^

a *P*<0.05 for T test or two-side X^2^ test for selected characteristics distributions between control and case groups.

### Statistical Analyses

Statistical analyses were carried out using Stata 10.0 statistical software package (Stata Corp, LP). Pearson’s chi-squared test was used to test the differences of categorical variables such as drinking and smoking status between cases and controls. Student’s t-test was used to test for differences in continuous variables such as age and body mass index (BMI) between groups. For SNP main effects analysis, we used additive genetic models. For exposure main effects analysis and interaction analysis, we categorized exposure variables ordinally (none/low/high). Men with urinary exposure level below the LOD were the reference group (non-exposed) and those with urinary exposure levels above the LOD were categorized into two groups using the urinary concentration median among samples with detectable levels as the cut-point separating “low” and “high”.

Associations between exposure and the risk of male infertility were evaluated by computing odds ratios (ORs) and their 95% confidence intervals (CIs) from logistic regression analyses with adjustment for age, BMI and urinary creatinine. The joint effects of exposure were evaluated by using multivariate logistic regression to calculate the odds of infertility associated with detection of multiple chemicals in urine, adjusted for age and BMI, with the non-exposed group as a reference. We used QVALUE software to calculate false discovery rate (FDR)-adjusted P value [25].

Potential gene-environment interactions were tested by comparing the changes in deviance (−2 log likelihood) between models of main effects with or without the interaction term. Stepwise regression was used to avoid interference and identify the most important interactions. We used the homozygous wild-type, non-exposed men as the reference category. The ORs and 95% CIs for the remaining genotype-exposure categories were estimated. An alpha of 0.05 was considered the threshold of significance.

## Results

### Characteristics of the Study Population

The study contained 589 infertile male patients and 396 control men proven fertility. No significant differences were identified between case and control groups with regard to smoking and drinking status. However, there were significant differences in age and BMI between cases and controls (Table 1). The mean (± SD) age and BMI were 29.75±3.43 years and 23.73±3.33 in control group, respectively. The mean (± SD) age and BMI in case group are 28.41±4.61 years and 23.26±3.19.

### Associations between Urinary Exposure Levels and Male Infertility

**Figure 1:**
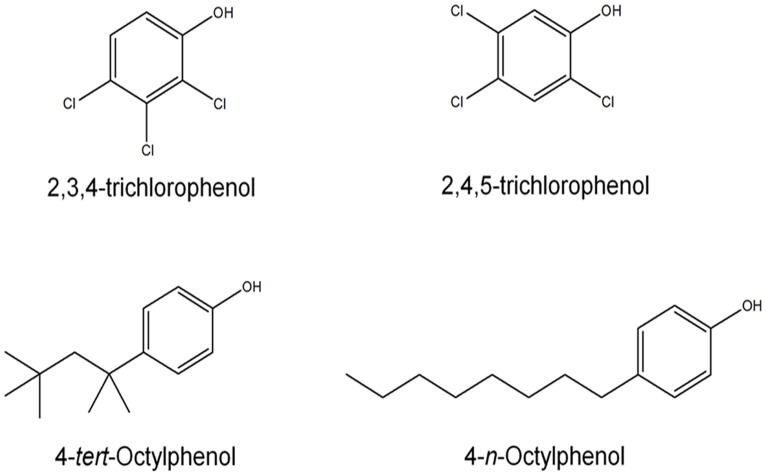
Molecular structures of 4-tert-Octylphenol (4-t-OP), 4-n-Octylphenol (4-n-OP), 2,3,4-Trichlorophenol (2,3,4,-TCP) and 2,4,5-Trichlorophenol (2,4,5-TCP).

**Figure 2:**
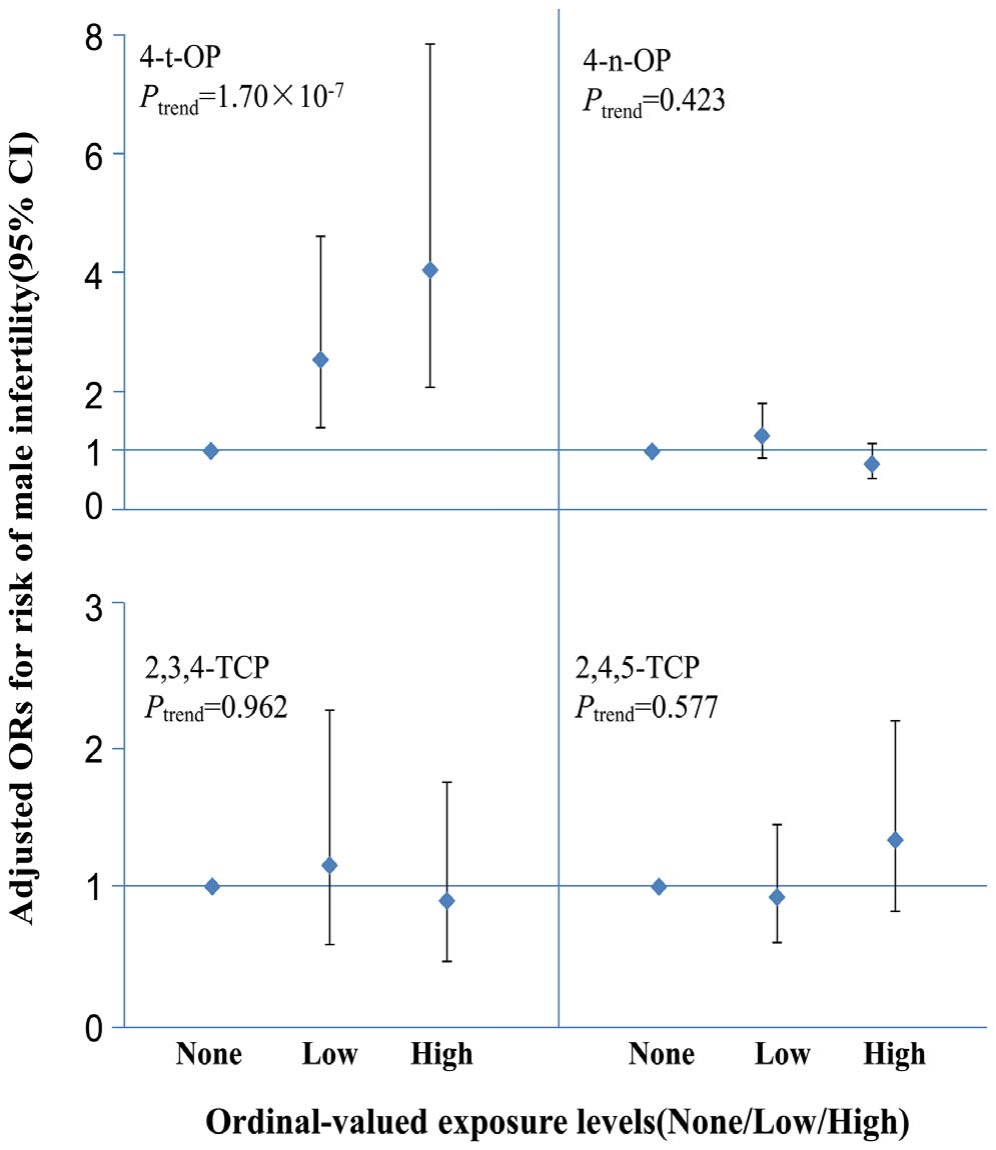
Adjusted ORs for risk of male infertility (95%CI). Logistic regression was used with categories of ordinal-valued exposure variables of 4-t-OP, 4-n-OP, 2,3,4-TCP and 2,4,5-TCP, adjusted for age, BMI and creatinine (n = 985).

The structure of 4-t-OP, 4-n-OP, 2,3,4-TCP, 2,4,5-TCP are presented in Figure 1, and distributions of their urinary concentrations in the 985 participants are presented in Table 2. Geometric means of 4-t-OP, 4-n-OP, 2,3,4-TCP and 2,4,5-TCP were 0.60 µg/L, 0.05 µg/L, 0.53 µg/L and 0.32 µg/L, respectively. There were no associations between 4-n-OP, 2,3,4-TCP, or 2,4,5-TCP and male infertility. High exposure to 4-t-OP was significantly associated with male infertility (*P*_trend_ = 1.70×10^−7^, OR = 4.05, 95% CI = 2.08–7.87) (Figure 2, Table S1, Table S2). Considering the possibility of combined risk from being exposed to multiple chemicals, we analyzed the joint effect of these four phenols on male infertility. We found that men exposed to more than one chemical had significantly elevated odds of infertility (*P*_trend_ = 0.008) (Table 3).

**Table 2:** Distribution of 4-t-OP, 4-n-OP, 2,3,4-TCP, and 2,4,5-TCP concentrations in urine (µg/L) in Han-Chinese males (n = 985).

Analytes	Geometricmean(95%CI)	percentile				
		50th	75th	90th	95th	99th
4-t-OP	0.60 (0.56–0.64)	< LOD	< LOD	0.46	0.65	1.32
4-n-OP	0.05 (0.05–0.06)	< LOD	0.03	0.08	0.11	0.45
2,3,4-TCP	0.53 (0.48–0.59)	< LOD	< LOD	< LOD	0.43	1.02
2,4,5-TCP	0.32 (0.29–0.35)	< LOD	< LOD	0.24	0.37	0.85

LOD for 4-t-OP, 4-n-OP, 2, 3, 4-TCP and 2, 4, 5-TCP were 0.34 µg/L, 0.02 µg/L, 0.28 µg/L, and 0.15 µg/L, respectively.

**Table 3:** Joint effects of urinary 4-t-OP, 4-n-OP, 2,3,4-TCP and 2,4,5-TCP Levels and male infertility (n = 985).

No. of exposed chemicals	Ca/Co	OR(95%CI)^a^	*P* b
Reference group	229/179	1.00(reference)	
1	243/163	1.26(0.94–1.69)	0.121
≥2	117/54	1.77(1.19–2.62)	0.010
***P*** **_trend_**			0.008

Ca: Cases; Co: Controls.

a Adjusted for age, BMI and creatinine.

b False Discovery Rate-corrected *P* value.

### Associations between Polymorphisms and Male Infertility

A total of 20 SNPs in metabolism enzyme genes were investigated in our study. Results for SNP main effects in relation to male infertility are presented in Table 4. However, no significant associations with male infertility were retained after FDR adjustment.

**Table 4:** Associations between metabolism enzyme gene SNPs and male infertility.

Gene	SNP	Position	Nucleotidechange	MAF^a^	OR(95%CI)b, c	*P* d
*CYP1A1*	rs1048943	nsSNP	A>G	0.256	1.08(0.52–2.25)	0.938
*CYP2B6*	rs3760657	5F	A>G	0.146	0.76(0.38–1.52)	0.938
*CYP2B6*	rs2054675	5F	T>C	0.185	1.73(0.76–3.98)	0.776
*CYP2B6*	rs707265	5F	G>A	0.341	0.96(0.63–1.45)	0.938
*CYP2B6*	rs1042389	3′UTR	T>C	0.314	0.93(0.58–1.51)	0.938
*CYP2C8*	rs1058932	3′UTR	C>T	0.372	1.11(0.72–1.71)	0.938
*CYP2C9*	rs4918758	5F	T>C	0.337	0.87(0.57–1.31)	0.938
*CYP2C19*	rs3814637	5F	C>T	0.116	1.06(0.29–3.92)	0.977
*CYP2C19*	rs4986894	5F	T>C	0.244	0.91(0.56–1.47)	0.938
*CYP2C19*	rs11568732	5F	T>G	0.093	2.46(0.66–9.19)	0.776
*CYP2S1*	rs3810171	5F	C>T	0.100	7.51(0.96–58.89)	0.730
*CYP2S1*	rs338583	3′UTR	T>C	0.195	1.00(0.46–2.19)	0.992
*NAT1*	rs7845127	5F	C>T	0.407	1.18(0.77–1.82)	0.938
*NAT1*	rs10888150	5F	T>C	0.442	0.95(0.65–1.39)	0.938
*NAT2*	rs1799930	nsSNP	G>A	0.167	1.17(0.56–2.42)	0.938
*NAT2*	rs1799931	nsSNP	G> A	0.222	0.65(0.28–1.51)	0.938
*NAT2*	rs4646246	5F	G>A	0.488	1.44(0.97–2.15)	0.730
*NAT2*	rs4646243	5F	C>T	0.411	0.76(0.51–1.12)	0.776
*SULT1E1*	rs4149525	5F	A> G	0.267	0.95(0.58–1.55)	0.938
*SULT1E1*	rs3736599	5F	G>A	0.226	1.04(0.79–1.36)	0.938

a Minimum allele frequency in the general Han Chinese population.

b Additive genetic model was used.

c Adjusted for age and BMI.

d False Discovery Rate-corrected *P* value.

### Interaction between Exposure Level and Polymorphisms on Male Infertility

We tested for interactions between 4-t-OP and metabolism enzyme gene polymorphisms. About twenty interactions that were tested met the FDR-adjusted *P* value <0.05 criterion in total (Table S2). We then used stepwise regression to avoid interferences and to identify the most important interactions. In the end, interactions between 4-t-OP and rs4918758, rs4986894 and rs1048943 were retained (Table 5). The strongest interaction was between 4-t-OP and rs4918758 in *CYP2C9* (*P*_inter_ = 6.05×10^−7^). Odds for infertility were significantly increased with increasing 4-t-OP exposure among men with TC/CC genotype (for low exposure level compared with non-exposed, OR = 2.26, 95%CI = 1.06, 4.83; for high exposure level compared with non-exposed, OR = 9.22, 95%CI = 2.78, 30.59; but no association among with TT genotype for low exposure level compared with non-exposed [OR = 2.56, 95%CI = 0.91, 7.16, for high exposure level compared with non-exposed; OR = 1.62, 95%CI = 0.67, 3.96]). We observed a significant interaction between rs4986894 in *CYP2C19* and 4-t-OP on male infertility only among the TC/CC genotype and high 4-t-OP exposure group (*P*_inter_ = 8.09×10^−7^, OR = 17.35, 95%CI = 2.33–129.04). The interaction between 4-t-OP exposure and rs1048943 in *CYP1A1* was also significant after adjustment (low level compared with non-exposed, OR = 2.00, 95%CI = 1.00, 4.00; for high level compared with non-exposed, OR = 3.44, 95%CI = 1.56, 7.57 in AA genotype group; for low level compared with non-exposed OR = 4.32, 95%CI = 1.23, 15.18; for high level compared with non-exposed, OR = 5.47, 95%CI = 1.59, 18.92 in AG/GG genotype).

**Table 5:** Interactions between urinary 4-t-OP, 4-n-OP, 2,3,4-TCP and 2,4,5-TCP levels and polymorphisms on male infertility.

Analytes	SNPs	Genotype	4-t-OP exposure level					
			None	Low		High		
4-t-OP			Ca/Co	Ca/Co	OR(95%CI)^a^	Ca/Co	OR(95%CI)^a^	*P*_inter_ b, c
*CYP2C9*	rs4918758	TT	163/115	19/7	2.56(0.91–7.16)	18/9	1.62(0.67–3.96)	6.05×10^−7^
		TC+CC	307/242	37/10	2.26(1.06–4.83)	43/4	9.22(2.78–30.59)	
*CYP2C19*	rs4986894	TT	230/153	26/9	2.06(0.86–4.96)	28/9	1.99(0.90–4.42)	8.09×10^−7^
		TC+CC	222/191	25/7	2.11(0.87–5.13)	30/2	17.35(2.33–129.04)	
*CYP1A1*	rs1048943	AA	291/228	35/13	2.00(1.00–4.00)	39/9	3.44(1.56–7.57)	3.73×10^−4^
		AG+GG	179/132	19/4	4.32(1.23–15.18)	21/4	5.47(1.59–18.92)	

None: non-exposed; Low: low level exposure; High: high level exposure; Ca: Cases; Co: Controls.

a Adjusted for age, BMI and creatinine.

b False Discovery Rate-corrected *P* value.

c *P*_inter_ for the stepwise regression <0.05.

## Discussion

Our study is the first study to evaluate the interaction between phenols exposure and metabolism enzyme polymorphisms in male infertility risk. In multivariable analyses for main effects, high exposure to 4-t-OP was associated with significant elevations in the odds of male infertility, while there were no significant associations between metabolism enzyme polymorphisms and male infertility. Three interactions were observed between4-t-OP and metabolism gene polymorphisms following adjustment.

OPs are widely used in industrial manufacturing and can be detected in the environment. Exposure of male rats to 4-t-OP caused increased sperm abnormalities and decreased sperm number [8]. 4-n-OP has also demonstrated estrogenic and antiandrogenic effects in vitro [7]. Due to the different structure of the branched alkyl chains, there are conflicting opinions about the toxicity of the 4-t-OP and 4-n-OP [26], [27]. Some groups have reported that 4-t-OP is the most estrogenic of the 4-alkylphenols, and in vitro studies have demonstrated that it binds to the estrogen receptor and activates estrogen-responsive genes [28], [29]. Sachiko Nomura used the rat liver to elucidate the metabolism of 4-t-OP and 4-n-OP depended on the shape of the alkyl chains. 4-t-OP is circulated between the liver and intestine, suggesting that a continuous exposure of the target organs to the chemical occurs [30]. This may be consistent with our results, where exposure to 4-t-OP, but not 4-n-OP, was found to be associated with increased odds of male infertility.

It is known that common diseases have complex etiologies related not only to genetic factors but also environmental factors. Recently, there has been increased interest in gene-environment interactions, which may affect infertility pathophysiology [31], [32]. Thus, we also studied the interactions between phenols exposure and the genetic variants in metabolism enzymes on male infertility. The most significant interaction was between 4-t-OP and rs4918758 in *CYP2C9* (*P*_inte_r = 6.05×10^−7^). It had a significant monotonic increase in male infertile risk with increasing 4-t-OP exposure in TC/CC genotype group, and no significant association in the TT genotype group. *CYP2C9*, which is a key member of CYP2C enzyme family, is responsible for the metabolism of drugs; genetic variants were reported to modify the enzyme activity, and subsequently cause toxicity [33]–[35]. Men carrying the rs4918758 minor allele may also have genetic susceptibility to male infertility risk when coupled with 4-t-OP exposure. We also found rs4986894 in *CYP2C19* combined with 4-t-OP exposure increased the odds of male infertility (*P*_inter_ = 8.09×10^−7^). This association was significant only among the men with the mutant genotype that were also highly exposed. *CYP2C19* is also a member of CYP2C enzyme family and can metabolize drugs. Rs4986894 is located in the promoter of *CYP2C19*, and may modulate the transcriptional activity of *CYP2C19*. For rs1048943 in *CYP1A1*, exposure to 4-t-OP in both genotype groups was associated with significantly increased odds of male infertility compared with the non-exposure group. *CYP1A1*, coding the enzyme aryl hydrocarbon hydroxylase, is believed to participate in the metabolism process of certain chemicals, which can induce bulky DNA adducts in human sperm, diminished semen quality, and may result in male infertility [36], [37]. Genetic variants in *CYP1A1* can influence the activity of this enzyme [38]. The rs1048943 polymorphism, an A to G substitution causing an Ile to Val amino acid exchange at codon 462, showed a significant higher catalytic activity for estrogens than the wild-type enzyme [38]. Niwa and colleagues reported that phenols exposure could inhibit the activity of CYPs, such as CYP2C9, *CYP2C19* and *CYP1A1* [20]. In vitro and in vivo studies also confirmed that OPs can alter the expression of steroidogenic enzymes through multiple mechanisms [39], [40]. Thus, we hypothesize that 4-t-OP exposure combined with polymorphisms in *CYP2C9*, *CYP2C19* and/or *CYP1A1* may interact to impart increased risk of male infertility. Our results could in part support this hypothesis.

Our study had a number of strengths. First of all, this is the first study to explore the potential interactions between phenols exposure and polymorphisms in metabolism enzymes on male infertility. Second, we used an improved UPLC-MS/MS method to detect the urinary OP levels, which was sensitive, accurate and easy to implement to ascertain our exposure data. In addition, we used FDR method and stepwise regression to adjust for multiple testing and reduce false-positive results. As with other case-control association studies, there were also several limitations to the present work. First, the interactions found in our study need to be confirmed using a larger study population and biologically through experimental research. Second, not all metabolism enzymes were included in this study and we may have missed some true interactions. Despite the limitations, our study, which had a large sample size, is the first to evaluate the interactions between OP exposure and gene polymorphisms on risk of male infertility.

### Conclusion

We found exposure to 4-t-OP could increase the male infertile risk and we also observed notable interactions between 4-t-OP exposure and metabolism enzyme gene polymorphisms in relation to risk of male infertility. Our study focuses on Han-Chinese men and will improve our understanding of male reproductive effects of 4-t-OP exposure. Future studies in larger populations are needed to replicate our findings, as are studies designed to explore the potential mechanisms involved.

## Supporting Information

Table S1 Associations between urinary 4-t-OP, 4-n-OP, 2,3,4-TCP, and 2,4,5-TCP levels and Male Infertility.
(DOCX)

Table S2 Interactions between urinary 4-t-OP, 4-n-OP, 2, 3, 4-TCP, 2, 4, 5-TCP levels and polymorphisms on Male Infertility.
(DOCX)
