# Supplementary material for: Interactions between Urinary 4-tert-Octylphenol Levels and Metabolism Enzyme Gene Variants on Idiopathic Male Infertility
Source: PLoS One. 2013 Mar 15;8(3):e59398. doi: 10.1371/journal.pone.0059398 (PMC3598701; doi:10.1371/journal.pone.0059398)
Supplement: Table S1 — Associations between urinary 4-t-OP, 4-n-OP, 2,3,4-TCP, and 2,4,5-TCP levels and Male Infertility. (DOCX) [file pone.0059398.s001.docx]

**Supplemental Table 1 Associations between urinary 4-t-OP, 4-n-OP, 2,3,4-TCP, and 2,4,5-TCP levels and Male Infertility.**

| **Analytes** | **None** | **Low** | | **High** | |  |
| --- | --- | --- | --- | --- | --- | --- |
|  | **Ca/Co** | **Ca/Co** | **OR(95%CI)^a^** | **Ca/Co** | **OR(95%CI)^a^** | ***P*_trend_** |
| **4-t-OP** | 472/366 | 56/17 | 2.54(1.40-4.63) | 61/13 | 4.05(2.08-7.87) | 1.70×10^-7^ |
| **4-n-OP** | 361/242 | 129/72 | 1.26(0.89-1.79) | 99/82 | 0.79(0.56-1.13) | 0.423 |
| **2,3,4-TCP** | 537/361 | 26/17 | 1.15(0.59-2.25) | 26/18 | 0.90(0.47-1.74) | 0.962 |
| **2,4,5-TCP** | 490/333 | 47/33 | 0.92(0.57-1.48) | 52/30 | 1.36(0.81-2.28) | 0.577 |

None: non-exposed; Low: low level exposure; High: high level exposure; Ca: Cases; Co: Controls.

^a^ Adjusted for age, BMI and creatinine.
