# Supplementary material for: Interactions between Urinary 4-tert-Octylphenol Levels and Metabolism Enzyme Gene Variants on Idiopathic Male Infertility
Source: PLoS One. 2013 Mar 15;8(3):e59398. doi: 10.1371/journal.pone.0059398 (PMC3598701; doi:10.1371/journal.pone.0059398)
Supplement: Table S2 — Interactions between urinary 4-t-OP, 4-n-OP, 2, 3, 4-TCP, 2, 4, 5-TCP levels and polymorphisms on Male Infertility. (DOCX) [file pone.0059398.s002.docx]

**Supplemental Table 2 Interactions between urinary 4-t-OP, 4-n-OP, 2, 3, 4-TCP, 2, 4, 5-TCP levels and polymorphisms on Male Infertility.**

| **Analyte** | **SNP** | **Genotype** | **4-t-OP, 4-n-OP, 2,3,4-TCP, 2,4,5-TCP exposure** | | | | |  |
| --- | --- | --- | --- | --- | --- | --- | --- | --- |
|  |  |  | **None** | **Low** | | **High** | |  |
| **4-t-OP** |  |  | **Ca/Co** | **Ca/Co** | **OR(95%CI)^a^** | **Ca/Co** | **OR(95%CI)^a^** | ***P***_inter_**^b^** |
| *CYP1A1* | rs1048943 | AA | 291/228 | 35/13 | 2.00(1.00-4.00) | 39/9 | 3.44(1.56-7.57) | 3.73×10^-4^ |
|  |  | AG+GG | 179/132 | 19/4 | 4.32(1.23-15.18) | 21/4 | 5.47(1.59-18.92) |  |
| *CYP2B6* | rs3760657 | AA | 317/242 | 34/13 | 1.93(0.96-3.88) | 31/10 | 2.84(1.27-6.37) | 2.31×10^-5^ |
|  |  | AG+GG | 154/118 | 22/4 | 5.13(1.49-17.68) | 29/3 | 7.14(2.13-23.93) |  |
| *CYP2B6* | rs2054675 | TT | 286/205 | 33/11 | 2.05(0.97-4.32) | 34/11 | 2.69(1.25-5.79) | 2.31×10^-5^ |
|  |  | TC+CC | 155/141 | 16/4 | 2.87(0.93-8.88) | 22/1 | 13.49(1.79-101.59) |  |
| *CYP2B6* | rs707265 | GG | 179/143 | 19/6 | 3.01(1.07-8.50) | 24/6 | 2.86(1.12-7.33) | 7.01×10^-5^ |
|  |  | GA+AA | 261/201 | 31/10 | 2.12(0.99-4.53) | 33/7 | 4.99(1.88-13.25) |  |
| *CYP2B6* | rs1042389 | TT | 203/180 | 25/5 | 5.39(1.82-16.01) | 23/7 | 3.63(1.33-9.89) | 4.48×10^-3^ |
|  |  | TC+CC | 214/160 | 24/12 | 1.51(0.70-3.23) | 28/6 | 3.73(1.49-9.33) |  |
| *CYP2C8* | rs1058932 | CC | 162/114 | 25/5 | 3.85(1.09-13.64) | 23/7 | 1.70(0.70-4.12) | 8.09×10^-7^ |
|  |  | CT+TT | 299/246 | 24/12 | 1.99(0.95-4.16) | 28/6 | 8.35(2.50-27.85) |  |
| *CYP2C9* | rs4918758 | TT | 163/115 | 19/7 | 2.56(0.91-7.16) | 18/9 | 1.62(0.67-3.96) | 6.05×10^-7^ |
|  |  | TC+CC | 307/242 | 37/10 | 2.26(1.06-4.83) | 43/4 | 9.22(2.78-30.59) |  |
| *CYP2C19* | rs3814637 | CC | 337/290 | 42/15 | 2.58(1.33-4.99) | 36/11 | 3.21(1.54-6.69) | 1.52×10^-2^ |
|  |  | CT+TT | 67/70 | 7/2 | 2.36(0.46-12.10) | 9/2 | 6.45(0.81-51.54) |  |
| *CYP2C19* | rs4986894 | TT | 230/153 | 26/9 | 2.06(0.86-4.96) | 28/9 | 1.99(0.90-4.42) | 8.09×10^-7^ |
|  |  | TC+CC | 222/191 | 25/7 | 2.11(0.87-5.13) | 30/2 | 17.35(2.33-129.04) |  |
| *CYP2C19* | rs11568732 | TT | 364/288 | 40/14 | 2.40(1.21-4.74) | 44/10 | 4.23(1.94-9.22) | 1.31×10^-2^ |
|  |  | TG+GG | 75/60 | 8/2 | 2.55(0.52-12.53) | 15/2 | 5.40(1.22-23.99) |  |
| *CYP2S1* | rs3810171 | CC | 297/252 | 37/14 | 2.51(1.26-5.00) | 47/10 | 4.70(2.16-10.23) | 3.47×10^-2^ |
|  |  | CT+TT | 171/111 | 19/3 | 3.86(1.10-13.48) | 13/3 | 3.49(0.96-12.60) |  |
| *CYP2S1* | rs338583 | TT | 315/248 | 35/13 | 2.04(1.02-4.06) | 39/7 | 5.02(2.07-12.13) | 1.02×10^-2^ |
|  |  | TC+CC | 144/107 | 19/4 | 4.64(1.32-16.30) | 18/5 | 3.16(1.04-9.63) |  |
| *NAT1* | rs7845127 | CC | 145/117 | 23/5 | 3.56(1.29-9.83) | 17/5 | 3.29(1.06-10.19) | 6.37×10^-5^ |
|  |  | CT+TT | 321/247 | 31/12 | 2.14(0.99-4.65) | 42/8 | 4.67(2.00-10.91) |  |
| *NAT1* | rs10888150 | TT | 147/122 | 18/5 | 4.44(1.24-15.95) | 21/2 | 7.56(1.71-33.31) | 7.20×10^-4^ |
|  |  | TC+CC | 297/234 | 34/12 | 2.03(0.99-4.16) | 37/11 | 3.34(1.53-7.29) |  |
| *NAT2* | rs1799930 | GG | 280/212 | 44/9 | 3.05(1.43-6.49) | 31/7 | 3.58(1.45-8.84) | 1.96×10^-3^ |
|  |  | GA+AA | 184/148 | 11/8 | 1.34(0.47-3.79) | 27/6 | 3.97(1.49-10.61) |  |
| *NAT2* | rs1799931 | GG | 330/258 | 37/13 | 2.23(1.09-4.54) | 50/11 | 3.74(1.84-7.59) | 2.38×10^-3^ |
|  |  | GA+AA | 122/103 | 17/4 | 3.08(1.00-9.44) | 11/2 | 7.41(0.94-58.60) |  |
| *NAT2* | rs4646246 | AA | 98/85 | 12/3 | 3.77(0.97-14.60) | 17/1 | 13.94(1.79-108.57) | 1.93×10^-4^ |
|  |  | AG+GG | 369/277 | 42/14 | 2.77(1.35-5.71) | 43/12 | 3.84(1.79-8.22) |  |
| *NAT2* | rs4646243 | CC | 122/88 | 14/6 | 1.36(0.49-3.78) | 15/1 | 9.54(1.22-74.49) | 2.31×10^-5^ |
|  |  | CT+TT | 314/259 | 36/11 | 2.61(1.17-5.82) | 41/11 | 3.14(1.43-6.89) |  |
| *SULT1E1* | rs4149525 | AA | 218/177 | 26/9 | 2.26(0.97-5.26) | 23/8 | 2.79(1.15-6.76) | 2.31×10^-5^ |
|  |  | AG+GG | 236/183 | 28/8 | 2.91(1.22-6.93) | 35/5 | 6.38(2.20-18.49) |  |
| *SULT1E1* | rs3736599 | GG | 230/175 | 25/11 | 1.89(0.87-4.11) | 26/5 | 3.64(1.35-9.79) | 2.31×10^-5^ |
|  |  | GA+AA | 236/187 | 31/5 | 4.67(1.59-13.71) | 34/8 | 4.23(1.72-10.41) |  |
| **4-n-OP** |  |  |  |  |  |  |  |  |
| *CYP1A1* | rs1048943 | AA | 216/151 | 85/45 | 1.36(0.88-2.11) | 64/54 | 0.84(0.54-1.30) | 2.97×10^-1^ |
|  |  | AG+GG | 140/88 | 44/26 | 1.28(0.72-2.25) | 35/26 | 0.86(0.48-1.54) |  |
| *CYP2B6* | rs3760657 | AA | 227/158 | 94/54 | 1.26(0.84-1.90) | 61/53 | 0.79(0.51-1.23) | 2.97×10^-1^ |
|  |  | AG+GG | 133/82 | 35/17 | 1.62(0.83-3.16) | 37/26 | 0.96(0.55-1.67) |  |
| *CYP2B6* | rs2054675 | TT | 218/143 | 71/44 | 1.02(0.65-1.61) | 64/40 | 1.00(0.63-1.60) | 2.89×10^-1^ |
|  |  | TC+CC | 113/85 | 48/25 | 1.51(0.85-2.68) | 32/36 | 0.56(0.32-0.96) |  |
| *CYP2B6* | rs707265 | GG | 138/92 | 47/27 | 1.33(0.74-2.40) | 37/36 | 0.69(0.39-1.21) | 3.37×10^-1^ |
|  |  | GA+AA | 191/135 | 74/41 | 1.20(0.74-1.94) | 60/42 | 0.89(0.55-1.46) |  |
| *CYP2B6* | rs1042389 | TT | 151/118 | 63/34 | 1.69(0.99-2.86) | 37/40 | 0.68(0.40-1.16) | 1.62×10^-1^ |
|  |  | TC+CC | 170/107 | 53/34 | 1.07(0.64-1.79) | 43/37 | 0.77(0.46-1.30) |  |
| *CYP2C8* | rs1058932 | CC | 116/81 | 44/21 | 1.37(0.74-2.54) | 40/25 | 0.96(0.52-1.75) | 2. 06×10^-1^ |
|  |  | CT+TT | 235/157 | 80/50 | 1.16(0.72-1.87) | 57/55 | 0.71(0.43-1.15) |  |
| *CYP2C9* | rs4918758 | TT | 122/88 | 41/21 | 1.29(0.70-2.40) | 37/22 | 1.02(0.55-1.89) | 1.95×10^-1^ |
|  |  | TC+CC | 237/149 | 88/49 | 1.31(0.82-2.10) | 62/58 | 0.73(0.45-1.16) |  |
| *CYP2C19* | rs3814637 | CC | 256/199 | 91/57 | 1.32(0.88-1.97) | 68/60 | 0.88(0.58-1.33) | 2.97×10^-1^ |
|  |  | CT+TT | 48/42 | 17/14 | 1.03(0.47-2.27) | 18/18 | 0.75(0.37-1.51) |  |
| *CYP2C19* | rs4986894 | TT | 173/113 | 61/29 | 1.36(0.80-2.32) | 50/29 | 0.98(0.57-1.68) | 2.97×10^-1^ |
|  |  | TC+CC | 168/117 | 62/39 | 1.09(0.67-1.80) | 47/44 | 0.69(0.42-1.13) |  |
| *CYP2C19* | rs11568732 | TT | 277/194 | 99/56 | 1.32(0.89-1.97) | 72/62 | 0.80(0.54-1.20) | 2.01×10^-1^ |
|  |  | TG+GG | 60/36 | 18/12 | 1.16(0.52-2.63) | 20/16 | 0.79(0.39-1.58) |  |
| *CYP2S1* | rs3810171 | CC | 227/161 | 85/53 | 1.13(0.75-1.72) | 69/62 | 0.68(0.45-1.03) | 2.01×10^-1^ |
|  |  | CT+TT | 131/81 | 43/17 | 1.80(0.95-3.41) | 29/19 | 1.28(0.66-2.48) |  |
| *CYP2S1* | rs338583 | TT | 244/167 | 79/49 | 1.15(0.75-1.77) | 66/52 | 0.87(0.57-1.34) | 3.17×10^-1^ |
|  |  | TC+CC | 104/71 | 45/20 | 1.76(0.97-3.21) | 32/25 | 0.83(0.46-1.50) |  |
| *NAT1* | rs7845127 | CC | 114/74 | 42/26 | 1.16(0.64-2.12) | 29/27 | 0.66(0.35-1.24) | 2.97×10^-1^ |
|  |  | CT+TT | 243/167 | 45/84 | 1.30(0.79-2.12) | 67/55 | 0.81(0.50-1.32) |  |
| *NAT1* | rs10888150 | TT | 108/80 | 44/19 | 1.90(1.00-3.63) | 34/30 | 0.86(0.48-1.56) | 2.97×10^-1^ |
|  |  | TC+CC | 229/157 | 77/52 | 1.16(0.72-1.89) | 62/48 | 0.97(0.59-1.60) |  |
| *NAT2* | rs1799930 | GG | 221/144 | 78/40 | 1.42(0.89-2.25) | 56/44 | 0.84(0.53-1.34) | 2.22×10^-1^ |
|  |  | GA+AA | 134/94 | 47/31 | 1.01(0.60-1.71) | 41/37 | 0.70(0.42-1.19) |  |
| *NAT2* | rs1799931 | GG | 259/170 | 94/55 | 1.17(0.78-1.76) | 64/57 | 0.73(0.48-1.12) | 3.30×10^-1^ |
|  |  | GA+AA | 88/68 | 31/17 | 1.28(0.66-2.50) | 31/24 | 0.85(0.48-1.53) |  |
| *NAT2* | rs4646246 | AA | 75/43 | 26/26 | 0.68(0.33-1.38) | 26/20 | 0.71(0.35-1.47) | 3.37×10^-1^ |
|  |  | AG+GG | 281/199 | 101/45 | 1.46(0.85-2.50) | 72/59 | 0.79(0.46-1.34) |  |
| *NAT2* | rs4646243 | CC | 88/55 | 37/17 | 1.52(0.75-3.08) | 26/23 | 0.73(0.37-1.44) | 2.97×10^-1^ |
|  |  | CT+TT | 241/174 | 83/54 | 1.02(0.62-1.70) | 67/53 | 0.79(0.47-1.33) |  |
| *SULT1E1* | rs4149525 | AA | 156/122 | 59/29 | 1.88(1.09-3.26) | 52/43 | 0.91(0.56-1.48) | 2.33×10^-1^ |
|  |  | AG+GG | 194/119 | 62/42 | 1.15(0.71-1.85) | 43/35 | 1.02(0.59-1.75) |  |
| *SULT1E1* | rs3736599 | GG | 169/114 | 59/36 | 1.16(0.70-1.91) | 53/41 | 0.90(0.55-1.47) | 2.95×10^-1^ |
|  |  | GA+AA | 186/126 | 69/34 | 1.49(0.90-2.46) | 46/40 | 0.75(0.46-1.25) |  |
| **2,3,4-TCP** |  |  |  |  |  |  |  |  |
| *CYP1A1* | rs1048943 | AA | 332/226 | 18/11 | 1.34(0.58-3.09) | 15/13 | 0.66(0.30-1.48) | 2.95×10^-1^ |
|  |  | AG+GG | 200/129 | 8/6 | 0.91(0.30-2.72) | 11/5 | 1.67(0.51-5.44) |  |
| *CYP2B6* | rs3760657 | AA | 343/242 | 20/11 | 1.43(0.65-3.15) | 19/12 | 0.91(0.42-1.96) | 3.06×10^-1^ |
|  |  | AG+GG | 192/113 | 6/6 | 0.83(0.23-2.94) | 7/6 | 1.13(0.32-3.95) |  |
| *CYP2B6* | rs2054675 | TT | 321/208 | 16/9 | 1.28(0.53-3.13) | 16/10 | 1.07(0.45-2.58) | 3.35×10^-1^ |
|  |  | TC+CC | 174/133 | 9/5 | 1.48(0.44-4.96) | 10/8 | 0.67(0.24-1.82) |  |
| *CYP2B6* | rs707265 | GG | 206/140 | 7/8 | 0.73(0.25-2.18) | 9/7 | 0.60(0.21-1.71) | 2.89×10^-1^ |
|  |  | GA+AA | 292/199 | 16/8 | 1.38(0.54-3.54) | 17/11 | 1.12(0.48-2.65) |  |
| *CYP2B6* | rs1042389 | TT | 231/173 | 15/9 | 1.22(0.49-3.02) | 5/10 | 0.34(0.10-1.17) | 2.89×10^-1^ |
|  |  | TC+CC | 241/163 | 9/8 | 1.03(0.37-2.87) | 16/7 | 1.35(0.53-3.42) |  |
| *CYP2C8* | rs1058932 | CC | 176/116 | 16/4 | 3.85(1.07-13.80) | 8/7 | 0.54(0.18-1.62) | 3.17×10^-1^ |
|  |  | CT+TT | 345/238 | 10/13 | 0.52(0.21-1.25) | 17/11 | 1.09(0.46-2.58) |  |
| *CYP2C9* | rs4918758 | TT | 177/116 | 11/6 | 1.26(0.45-3.55) | 12/9 | 0.61(0.24-1.56) | 2.97×10^-1^ |
|  |  | TC+CC | 358/236 | 15/11 | 1.00(0.41-2.43) | 14/9 | 1.20(0.46-3.11) |  |
| *CYP2C19* | rs3814637 | CC | 376/284 | 17/16 | 0.91(0.43-1.93) | 22/16 | 0.89(0.44-1.80) | 1.95×10^-1^ |
|  |  | CT+TT | 71/77 | 4/1 | 3.68(0.39-35.07) | 2/2 | 1.70(0.14-20.03) |  |
| *CYP2C19* | rs4986894 | TT | 254/155 | 17/6 | 1.99(0.75-5.27) | 13/10 | 0.64(0.26-1.61) | 3.15×10^-1^ |
|  |  | TC+CC | 256/184 | 9/9 | 0.64(0.22-1.83) | 12/7 | 1.09(0.40-3.01) |  |
| *CYP2C19* | rs11568732 | TT | 405/281 | 20/15 | 1.05(0.51-2.20) | 23/16 | 0.86(0.43-1.71) | 2.01×10^-1^ |
|  |  | TG+GG | 91/61 | 4/1 | 3.74(0.39-35.79) | 3/2 | 2.17(0.22-21.48) |  |
| *CYP2S1* | rs3810171 | CC | 348/248 | 17/13 | 1.20(0.54-2.65) | 16/15 | 0.68(0.31-1.49) | 2.55×10^-1^ |
|  |  | CT+TT | 185/110 | 8/4 | 1.14(0.32-3.99) | 10/3 | 2.19(0.59-8.17) |  |
| *CYP2S1* | rs338583 | TT | 351/244 | 22/11 | 1.58(0.72-3.47) | 16/13 | 0.85(0.37-1.89) | 2.89×10^-1^ |
|  |  | TC+CC | 168/105 | 4/6 | 0.50(0.13-1.93) | 9/5 | 1.02(0.32-3.21) |  |
| *NAT1* | rs7845127 | CC | 168/114 | 11/5 | 1.97(0.60-6.41) | 6/8 | 0.57(0.19-1.78) | 3.30×10^-1^ |
|  |  | CT+TT | 359/245 | 15/12 | 0.92(0.40-2.12) | 20/10 | 1.22(0.52-2.85) |  |
| *NAT1* | rs10888150 | TT | 161/119 | 12/3 | 2.96(0.81-10.89) | 13/7 | 1.64(0.56-4.79) | 1.30×10^-1^ |
|  |  | TC+CC | 344/232 | 12/14 | 0.73(0.31-1.74) | 12/11 | 0.64(0.26-1.56) |  |
| *NAT2* | rs1799930 | GG | 317/209 | 20/10 | 1.42(0.62-3.24) | 18/9 | 1.36(0.57-3.21) | 1.00×10^-1^ |
|  |  | GA+AA | 209/146 | 6/7 | 0.71(0.22-2.27) | 7/9 | 0.36(0.11-1.13) |  |
| *NAT2* | rs1799931 | GG | 377/260 | 19/10 | 1.73(0.73-4.08) | 21/12 | 1.07(0.50-2.31) | 1.02×10^-1^ |
|  |  | GA+AA | 141/96 | 5/7 | 0.39(0.11-1.36) | 4/6 | 0.48(0.12-1.85) |  |
| *NAT2* | rs4646246 | AA | 122/77 | 2/8 | 0.16(0.03-0.83) | 3/4 | 0.32(0.06-1.84) | 2.23×10^-1^ |
|  |  | AG+GG | 407/280 | 24/9 | 2.08(0.87-4.98) | 23/14 | 1.07(0.49-2.32) |  |
| *NAT2* | rs4646243 | CC | 127/90 | 13/2 | 4.32(0.94-19.85) | 11/3 | 2.57(0.69-9.56) | 2.83×10^-2^ |
|  |  | CT+TT | 370/252 | 10/15 | 0.52(0.21-1.29) | 11/14 | 0.46(0.18-1.16) |  |
| *SULT1E1* | rs4149525 | AA | 243/175 | 12/10 | 1.12(0.44-2.83) | 12/9 | 0.93(0.36-2.40) | 3.35×10^-1^ |
|  |  | AG+GG | 272/180 | 14/7 | 1.41(0.54-3.69) | 13/9 | 0.93(0.37-2.33) |  |
| *SULT1E1* | rs3736599 | GG | 254/175 | 13/7 | 1.30(0.47-3.57) | 14/9 | 1.07(0.43-2.63) | 3.30×10^-1^ |
|  |  | GA+AA | 276/183 | 13/10 | 1.11(0.46-2.71) | 12/7 | 0.90(0.33-2.45) |  |
| **2,4,5-TCP** |  |  |  |  |  |  |  |  |
| *CYP1A1* | rs1048943 | AA | 312/210 | 25/22 | 0.71(0.38-1.31) | 28/18 | 1.16(0.60-2.25) | 1.95×10^-1^ |
|  |  | AG+GG | 174/117 | 22/11 | 1.26(0.59-2.68) | 23/12 | 1.52(0.67-3.44) |  |
| *CYP2B6* | rs3760657 | AA | 325/220 | 26/22 | 0.82(0.45-1.51) | 31/23 | 1.01(0.55-1.87) | 9.64×10^-2^ |
|  |  | AG+GG | 163/108 | 21/11 | 1.15(0.53-2.46) | 21/6 | 2.86(1.05-7.78) |  |
| *CYP2B6* | rs2054675 | TT | 291/189 | 27/22 | 0.72(0.39-1.31) | 35/16 | 1.46(0.75-2.82) | 1.95×10^-1^ |
|  |  | TC+CC | 158/125 | 18/8 | 1.47(0.62-3.50) | 17/13 | 1.12(0.48-2.64) |  |
| *CYP2B6* | rs707265 | GG | 185/126 | 18/15 | 0.71(0.34-1.49) | 19/14 | 0.95(0.44-2.08) | 1.00×10^-1^ |
|  |  | GA+AA | 268/189 | 26/15 | 1.12(0.56-2.24) | 31/14 | 1.64(0.78-3.44) |  |
| *CYP2B6* | rs1042389 | TT | 207/162 | 24/14 | 1.23(0.61-2.49) | 20/16 | 0.92(0.43-1.97) | 7.72×10^-2^ |
|  |  | TC+CC | 216/150 | 20/17 | 0.78(0.39-1.57) | 30/11 | 2.42(1.10-5.33) |  |
| *CYP2C8* | rs1058932 | CC | 170/107 | 13/9 | 0.99(0.40-2.43) | 17/11 | 0.97(0.41-2.27) | 2.22×10^-1^ |
|  |  | CT+TT | 306/220 | 17/11 | 0.79(0.44-1.43) | 33/18 | 1.36(0.69-2.69) |  |
| *CYP2C9* | rs4918758 | TT | 168/114 | 11/8 | 0.95(0.36-2.49) | 21/9 | 1.62(0.68-3.87) | 2.97×10^-1^ |
|  |  | TC+CC | 321/214 | 36/24 | 0.93(0.52-1.67) | 30/18 | 1.24(0.63-2.45) |  |
| *CYP2C19* | rs3814637 | CC | 339/263 | 39/27 | 1.09(0.64-1.86) | 37/26 | 1.29(0.72-2.31) | 2.22×10^-1^ |
|  |  | CT+TT | 69/65 | 6/6 | 0.76(0.24-2.44) | 8/3 | 2.07(0.53-8.02) |  |
| *CYP2C19* | rs4986894 | TT | 237/146 | 19/14 | 0.79(0.38-1.66) | 28/11 | 1.53(0.71-3.30) | 3.12×10^-1^ |
|  |  | TC+CC | 230/165 | 27/18 | 0.84(0.44-1.61) | 20/17 | 0.93(0.43-2.00) |  |
| *CYP2C19* | rs11568732 | TT | 375/262 | 38/25 | 1.02(0.59-1.76) | 35/25 | 1.18(0.65-2.14) | 2.22×10^-1^ |
|  |  | TG+GG | 80/55 | 7/6 | 0.81(0.27-2.47) | 11/3 | 2.48(0.68-9.12) |  |
| *CYP2S1* | rs3810171 | CC | 313/238 | 33/19 | 1.27(0.69-2.32) | 35/19 | 1.73(0.91-3.29) | 1.95×10^-1^ |
|  |  | CT+TT | 173/93 | 14/14 | 0.71(0.33-1.55) | 16/10 | 1.09(0.46-2.57) |  |
| *CYP2S1* | rs338583 | TT | 322/224 | 29/26 | 0.73(0.41-1.29) | 38/18 | 1.77(0.94-3.33) | 3.17×10^-1^ |
|  |  | TC+CC | 150/97 | 17/7 | 1.66(0.67-4.13) | 14/12 | 0.82(0.33-2.01) |  |
| *NAT1* | rs7845127 | CC | 158/105 | 14/13 | 0.67(0.30-1.51) | 13/9 | 0.91(0.36-2.34) | 1.51×10^-1^ |
|  |  | CT+TT | 323/226 | 33/20 | 1.05(0.56-1.96) | 38/21 | 1.51(0.79-2.90) |  |
| *NAT1* | rs10888150 | TT | 149/109 | 20/12 | 1.15(0.53-2.48) | 17/8 | 1.93(0.76-4.92) | 2.97×10^-1^ |
|  |  | TC+CC | 311/216 | 25/21 | 0.85(0.44-1.62) | 32/20 | 1.41(0.71-2.77) |  |
| *NAT2* | rs1799930 | GG | 300/194 | 24/19 | 0.71(0.37-1.36) | 31/15 | 1.89(0.92-3.91) | 2.01×10^-1^ |
|  |  | GA+AA | 183/133 | 21/14 | 1.01(0.49-2.06) | 18/15 | 0.71(0.33-1.53) |  |
| *NAT2* | rs1799931 | GG | 345/236 | 33/27 | 0.79(0.45-1.36) | 39/19 | 1.53(0.83-2.81) | 2.95×10^-1^ |
|  |  | GA+AA | 126/93 | 13/6 | 1.43(0.53-3.88) | 11/10 | 1.06(0.38-2.99) |  |
| *NAT2* | rs4646246 | AA | 102/72 | 14/9 | 1.21(0.49-3.00) | 11/8 | 0.75(0.26-2.16) | 2.01×10^-1^ |
|  |  | AG+GG | 381/257 | 33/24 | 0.92(0.49-1.72) | 40/22 | 1.77(0.91-3.42) |  |
| *NAT2* | rs4646243 | CC | 123/81 | 11/10 | 0.63(0.25-1.57) | 17/4 | 3.73(1.05-13.31) | 2.97×10^-1^ |
|  |  | CT+TT | 326/237 | 34/22 | 1.01(0.54-1.88) | 31/22 | 1.06(0.54-2.10) |  |
| *SULT1E1* | rs4149525 | AA | 216/166 | 25/16 | 1.14(0.58-2.24) | 26/12 | 1.83(0.86-3.90) | 3.49×10^-1^ |
|  |  | AG+GG | 255/163 | 20/16 | 0.91(0.45-1.85) | 24/17 | 1.33(0.63-2.78) |  |
| *SULT1E1* | rs3736599 | GG | 235/165 | 21/11 | 1.27(0.58-2.76) | 25/15 | 1.33(0.65-2.72) | 2.97×10^-1^ |
|  |  | GA+AA | 249/164 | 26/22 | 0.80(0.43-1.47) | 26/14 | 1.60(0.74-3.48) |  |

None: non-exposed; Low: low level exposure; High: high level exposure; Ca: Cases; Co: Controls.

^a^ Adjusted for age, BMI and creatinine. ^b^ False Discovery Rate-corrected *P* value.
